# Personalized risk score prediction and testing policy adaptations of a COVID-19 population-based contact tracing network

**DOI:** 10.1017/S0950268825100319

**Published:** 2025-07-24

**Authors:** Shushan Wu, Yan Feng, Huimin Cheng, Hui Huang, Yang Li, Feng Ling, Ping Ma, Wenxuan Zhong, Ye Shen

**Affiliations:** 1 Department of Statistics, University of Georgia, Athens, GA, USA; 2 Zhejiang Provincial Center for Disease Control and Prevention, Hangzhou, China; 3 Department of Biostatistics, Boston University, Boston, MA, USA; 4 School of Statistics, Renmin University of China, Beijing, China; 5 Department of Epidemiology and Biostatistics, College of Public Health, University of Georgia, Athens, GA, USA; 6 Institute of Health Data Science, Renmin University of China

**Keywords:** COVID-19 contact tracing, graph neural network, personalized risk score, PCR testing policy, semi-supervised

## Abstract

Contact tracing is an effective public health policy to put the fast-spreading epidemic under control. The government tracks the contacts of confirmed SARS-CoV-2 cases, recommends testing, encourages self-quarantine, and monitors symptoms of contacts. In developing and less-developed countries with limited resources for widespread SARS-CoV-2 testing, it remains essential to identify and quarantine positive contacts to control outbreaks. Therefore, analysing recall and precision when implementing testing policies for these contacts is necessary. We analysed a contact tracing dataset from a cohort of 827 index patients infected with SARS-CoV-2 and their 14814 close contacts from Jan 2020 to July 2020 in a province in eastern China. We constructed a network from the data and used a Graph Convolutional Network to predict each contact’s infection status. To the best of our knowledge, this is the first method to use population-based contact tracing data for predicting the infection status using graph neural networks. Despite limited information, our model achieves competitive Area Under the Receiver Operating Characteristic Curve (ROC AUC) compared to hospital-onset scenarios. Based on the risk scores, we propose several contact testing policy adaptations that balance resource efficiency and effective pandemic control.

## Introduction

Contact tracing is an effective public health policy for controlling fast-spreading epidemics [1–3]. For example, during the COVID-19 pandemic, several governments implemented contact tracing programs. In these cases, once an infectious patient is identified, the government promptly tracks their contacts, refers them for SARS-CoV-2 testing, encourages self-quarantine, and monitors their symptoms.

**Figure 1.**
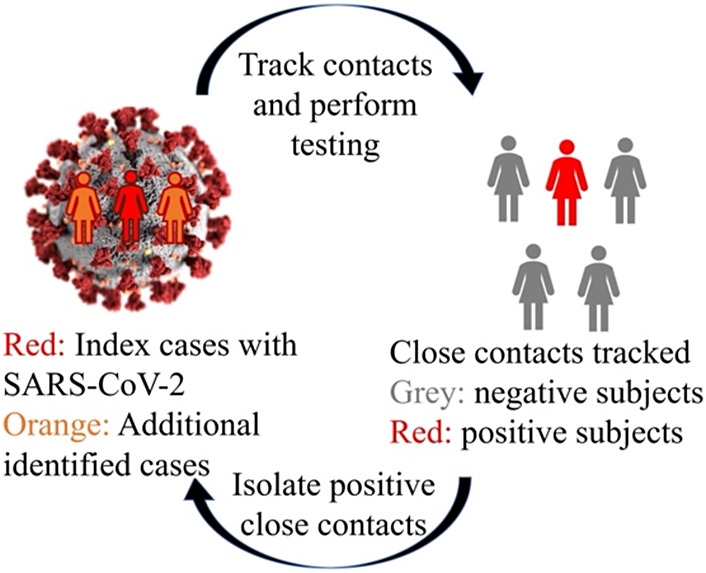
An illustration of a typical contact tracing program. Contacts were defined as individuals who had direct or indirect interactions with confirmed COVID-19 cases. Close contacts were quarantined for at least 14 days, either centrally or at home if resources were limited. Health professionals monitored symptoms daily, and SARS-CoV-2 tests were administered if respiratory symptoms arose or if a physician suspected infection. If a contact tested positive, contact tracing was initiated for their contacts.

Figure 1 illustrates a typical contact tracing program. To break the transmission chain, contact tracing aims to identify as many exposed individuals (infected but not yet infectious) and infectious patients as possible during the quarantine period and then isolate them to contain the outbreak. After conducting SARS-CoV-2 testing on all contacts of the index cases, the government obtains the testing results for close contacts. Positive cases, referred to as additional identified cases, require further tracking of their close contacts.

In some countries, legal restrictions make it challenging to enforce mandatory SARS-CoV-2 testing and quarantine for close contacts. Moreover, limited early-stage testing capacity and human resources [4, 5] have resulted in a significant proportion of contacts’ test results remaining elusive. Most contact tracing data is based on patients in hospitals or clinical settings [6–11]. Instead, we use population-cohort contact tracing data from one province in China. Most methods use a logistic regression model to predict, which does not use network structure [7–9]. Some of the methods use the XGBoost model [5, 10] or Gradient Boosting [6] to predict risk scores. Although Myall et al. [10] used some network-summarized statistics, they do not utilize the local information in the graph and sometimes this leads to information loss in prediction. Furthermore, we extend our results to suggest that public policymakers adapt contact tracing policies, balancing resources and efficiency. As far as we know, there is no discussion yet regarding public policies based on the prediction of risk scores, except for some interpreting the model by finding the key clinical predictors of the severe syndrome of COVID-19 [11]. Most existing studies have developed prediction models based on hospitalized patients, relying on clinical information as predictors. These models have limited applicability to population-based contact tracing cohorts. Few studies have used contact tracing data not limited to the clinical settings [12]. Huang et al. have identified hidden spreaders by modelling the disease transmission probability using a Markovian process [12]. The disease transmission probability is influenced by factors such as the nature of the contact, duration of exposure, and the infectiousness of the disease, whereas the contacts’ demographic information is not considered. To surmount the challenges, we aim to predict the infection risk scores of these untested close contacts using population-based contact tracing data with limited information.

Moreover, traditional prediction methods [13, 14] fail to leverage network structure information, leading to unsatisfactory prediction outcomes. This limitation drives us to propose a more accurate node classification model that predicts missing testing results and assesses the risk of each contact. These improvements could assist local governments in refining their contact tracing programs [15].

Local governments’ contact tracing efforts are influenced by numerous factors, making comprehensive tracing unrealistic. As a result, it is essential to focus on testing only a subset of contacts and quarantining those at highest risk to conserve resources and effectively mitigate the outbreak [5]. To address this need, we propose several adaptations to the current contact tracing surveillance programme, prioritizing high-risk close contacts for testing and quarantine.

## Methods

### Data design and setting

We obtained a population-based cohort of 827 index patients infected with SARS-CoV-2 and their 14814 close contacts under a contact tracing surveillance programme from January 2020 to July 2020 from a province in eastern China. The field workers documented their contact process information, demographics, SARS-CoV-2 testing results, and severity levels if infected for each subject. Using this dataset, we constructed a contact tracing network with 15641 nodes and 15246 edges, where each node represents either a contact or a case, and each edge represents those two persons who had contact before. Additionally, each edge is weighted using contact time and contact type, and each node has an attribute (feature) vector containing a person’s demographics, neighbourhood information about cases, contact types, SARS-CoV-2 test results, and symptom severity levels. Among the 14814 contacts, 5946 did not take any SARS-CoV-2 test due to the lack of test kits in the early stage of the pandemic [16].

**Figure 2.**
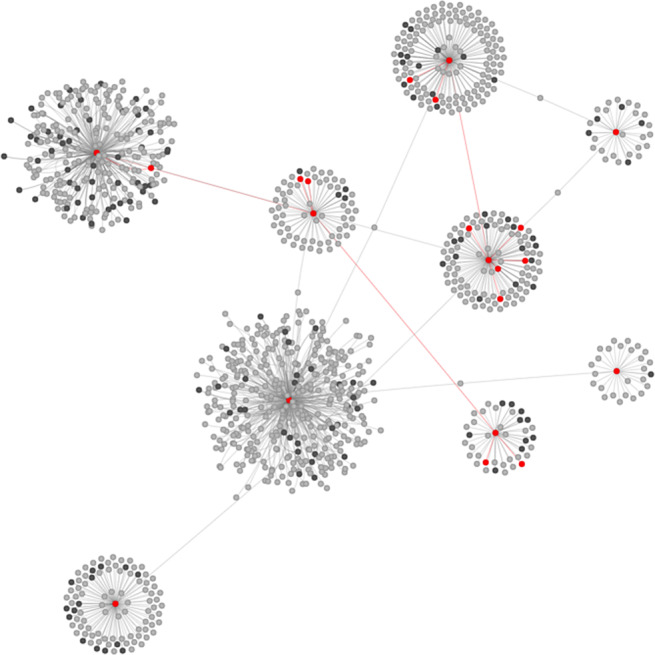
A visualization of the largest component of the whole contact tracing network. The grey nodes are negative subjects. The red nodes are positive subjects. The black nodes are subjects whose status is unknown.

**Figure 3.**
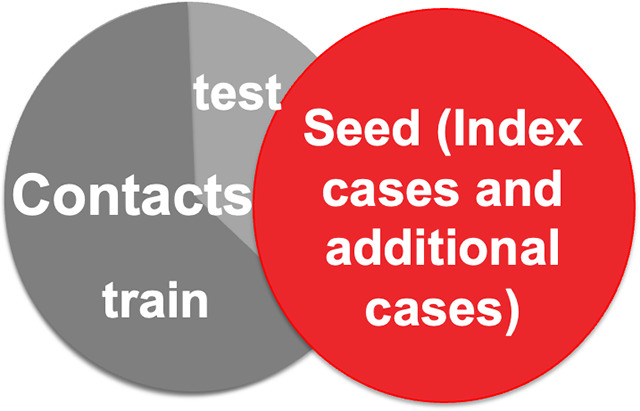
Dataset Composition. The seed nodes are defined by coupling the index cases and additional identified cases from the contact tracing program. The contact nodes are divided into 60% for the training dataset and 40% for testing.

As shown in Figure 2, the red nodes are positive subjects, including the index cases and additional identified cases, and grey nodes are negative subjects. Black nodes are subjects whose status is unknown and about 6000 contacts’ PCR testing results are missing. We define seed nodes by coupling index cases and additional identified cases from the contact tracing programme. Next, we need to train and evaluate our model for the remaining close contacts based on their information. Here, we divide the contact nodes into 60% for the training dataset and 40% for the testing dataset as shown in Figure 3.

### Model variables

We classified the features into three sets. (i) Demographics of subjects, including age and gender information. (ii) Neighbours’ information, including the number of affected cases among neighbours, the maximum age of the cases among the neighbours, and the most severe level of infection of the cases among the neighbours. (iii) Contact type information, including the number of contacts who are living together and the number of contacts who dine together. More details are given in the section “Overview of the Dataset” in the Supplementary Materials.

### Risk score prediction framework

**Figure 4.**
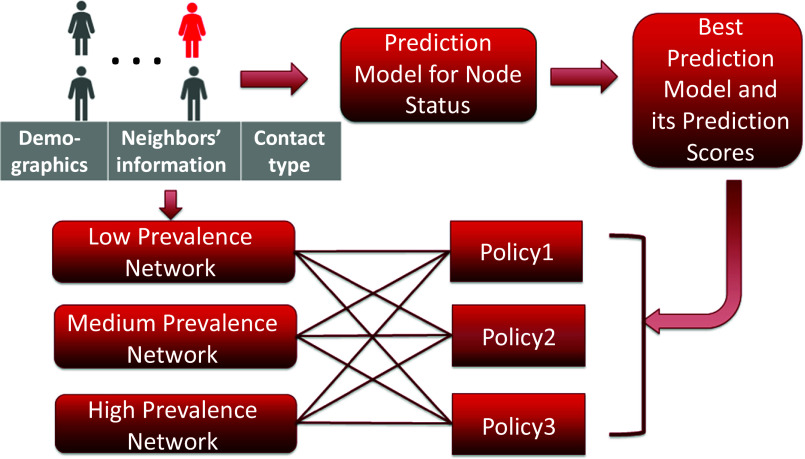
Framework of analysis.

The contact tracing data is intrinsically network structured. After obtaining the information collected by the field workers, we assign to every node a feature vector listed in ‘Model Variables’. We use the framework shown in Figure 4 to analyse the contact tracing data to achieve our goals. The input of the prediction model comprises all combinations of the three feature sets. As we obtain PCR testing results for only a fraction of the contacts, and the data is intrinsically network structured, we will utilize the Graph Convolutional Network (GCN) for predicting [17]. The GCN can exploit the network structure given by the contact tracing network if the connected nodes in the graphs are likely to share the same label. We also construct prediction models using other traditional classification models, such as the Logistic Regression Model (LR) [14] and the Random Forest Model (RF) [13]. Then, we select the best model to obtain the risk scores for each contact.

After the prediction for each contact, we can also get the prediction scores for each contact. Based on the prediction score, also called personalized risk scores, the government could apply different policies after evaluating the local epidemiological condition and financial availabilities. Therefore, we suggest three sampling testing policies for the contacts tracked to save resources instead of comprehensively testing for all other close contacts.

### Methods for prediction of node status

In this study, most nodes in the contact tracing network do not have labels. To predict the personalized risk scores of a subject, we can utilize the information of the subject’s neighbours and the contact information other than relying on the information of the subject alone. For example, if we ignore the network information, we can use only the demographic data and the node features; subjects with more similar demographics and node features tend to have the same testing results and thus, more similar personalized risk scores.

However, this is not the case when two subjects have similar node features but different neighbourhood information. For example, if the subject is located in a neighbourhood with higher infection-prevalence levels, the subject would have higher personalized risk scores than the subject located in a lower infection-prevalent neighbourhood. In addition to the contact type, the contact time also impacts the personalized risk scores. We then weight each edge in the network by the contact time, wherein the longer the contact time, the larger the edge weight is. We assume that when the contact event has a longer contact time, the subjects are more susceptible to infection, and thus the personalized risk score is higher.

### Graph convolutional neural network

The network data can be abstracted as a graph, 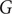 , where the connectivity information can be formatted as an adjacency matrix. Each row or column contains the connectivity information of each node in the graph, and each entry in an adjacency matrix is the weight of the edge that connects the two nodes. The adjacency matrix can be denoted as 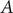 , and the node feature matrix can be denoted as 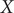 , where each row corresponds to each node’s feature vector. Our goal is to predict the labels of each node, 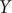 , and obtain the probability of the subject being tested positive, that is, 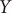 being 1.

The graph convolutional network (GCN) first learns the embedding of each node and uses embedding for the downstream task, e.g., semi-supervised node classification. The graph convolution layer can pass messages from the neighbourhood information in accordance with the following layer-wise propagation rule:

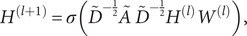

where 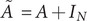 is the adjacency matrix of 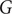 with self-loops. 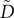 is the degree matrix where the diagonal values are the sum of neighboring edge weights of each node, 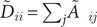 , 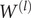 is the weight matrix to be trained in the 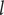 -th layer, 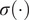 denotes an activation function, and 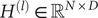 is the embeddings learned after 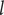 convolutions of dimension 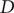 . When 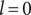 , 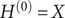 . For the nodes that are not directly connected to each other but can be reached by traversing two or more edges, we can call them multi-hop neighbors. By applying several graph convolution layers, the model can learn node embeddings by integrating features from neighbors multiple hops away.

### Semi-supervised node classification

One challenge in the personalized risk score prediction is that not all the nodes in the graph have labels as not all the traced contacts took the SARS-CoV-2 tests due to lack of test kits. However, each subject in the contact tracing network has node features, including the demographics, neighbours’ information, and contact type information. This poses a challenge in leveraging all the feature vectors of the subjects, when only a fraction of the subjects has the PCR test results. We consider a two-layer GCN for the semi-supervised node-classification task. The adjacency matrix 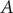 is weighted. Let 
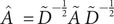
 , and the model of predicting the personalized risk score is:

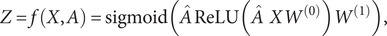

where the sigmoid function is to predict the binary outcome, defined as 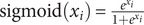 , and 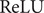 is the activation function in the first convolution layer, defined as 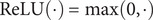 . The weight matrices 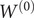 and 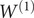 are trainable. For the semi-supervised binary classification task, the objective function is the cross-entropy loss over all the labelled examples:

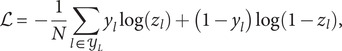

Where 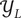 is the set of node indices that have labels. For training the GCN, we use the full dataset for each training iteration.

We compared three different classification models: Logistic Regression, Random Forest, and Graph Convolutional Neural Network. Owing to information loss and data privacy issues, information retrieved from most of the contacts is only the age and gender. Therefore, we would like to compare different models under different combinations of feature sets.

For the comparison, we used the AUC [18] score for the ROC for evaluating; then we select the best model to obtain the prediction score for each of the close contacts, that is, the personalized risk scores. Given the risk score, we can then suggest adopting the relevant contact tracing programs by local governments [15].

### Risk score-based testing policies

Based on the personalized risk scores, we can obtain the risk of being infected in the case of each contact and then we propose sampling policies to test tracked contacts. (i) The first policy is to screen contacts with the highest risk scores based on the budget allocation. (ii) The second policy uses personalized risk scores to generate probabilities to sample close contacts. (iii) The third policy considers the local area’s prevalence level, generating probability combining the prevalence level of components and risk scores.

Also, we evaluate the performance of the proposed policies under different simulated settings, including different prevalence levels and available budget allocations to perform testing. There are 653 components associated with the contacts in the whole contact tracing network. Each component has a prevalence level (low, medium, and high) according to its proportion of positive subjects. We then bootstrap [19] the components and obtain the simulated low-prevalence network, medium-prevalence network, and high-prevalence network. We design three SARS-CoV-2 testing policies for the contacts under varying budget proportions for each prevalence level network. More details about the generation of the simulated data and policy information is provided in the Algorithm section in the Supplementary Materials.

**Figure 5.**
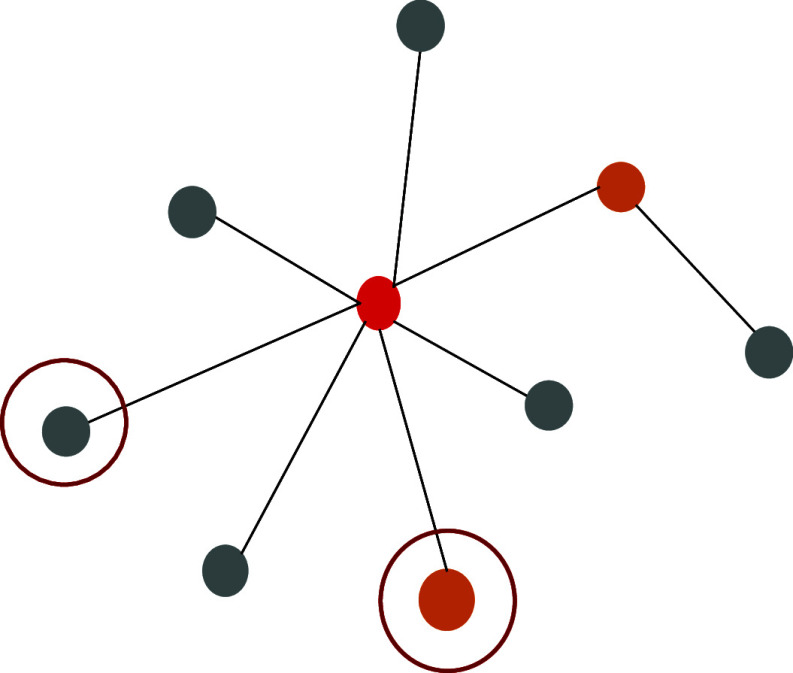
An illustration of a component in medium prevalence. The red nodes are index cases, the orange nodes are positive contacts, and the grey nodes are negative contacts. The two circled nodes are the suspected nodes that we selected by our proposed policy.

We expect the selected close contacts to be more likely to be positive and more infective. Thus, the quarantine and the contact tracing afterwards would be more effective during the entire transmission process. Here, we used pseudo-precision and pseudo-recall to evaluate our SARS-CoV-2 testing policies. To better comprehend how we calculate the evaluation metrics of different testing policies, we show a small example in Figure 5.

Figure 5 shows an illustration of one connected component. The red node is the index case in the component; the two orange nodes are positive contacts; the rest are negative contacts; the two circled nodes are suspected (predicted positive) contacts selected or sampled by a given testing strategy. The prevalence level in this example is 3/9 = 1/3, which is medium. The number of positive contacts of suspicious nodes (the number of true positive) is 1; and the number of positive contacts in this component is 2. The ratio of positive contacts we screened out is calculated by 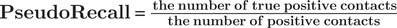 , which is 50%; the positive predictive value is calculated by 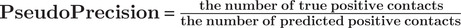 , which is 50%.

## Results

We compared different models’ prediction accuracies, given the different combinations of feature sets [20]. The goal of comparing the models (GCN, LR, RF) and feature sets is to identify the optimal combination of the model and the feature set that delivers the best performance. This will guide us in the development of screening policies based on the predicted personalized risk scores. Based on the results given in Table 1, we can also assess the contribution of various types of information (feature sets 1 to 3 and network structure) to the prediction task.

**Table 1.** The AUC scores of different prediction models with different feature sets

AUC-ROC	GCN	LR	RF
Feature Set 1	0.74(0.71–0.77)	0.59(0.56–0.62)	0.56(0.53–0.59)
Feature Set 1&2	0.74(0.70–0.78)	0.65(0.61–0.69)	0.71(0.68–0.74)
Feature Set 1&3	0.845(0.82–0.87)	0.75(0.72–0.78)	0.81(0.83–0.78)
All Feature Sets	0.84(0.80–0.88)	0.73(0.69–0.76)	0.82(0.78–0.84)

*Note*: The numbers in the brackets are the range of AUC scores for 30 times of splitting the dataset.

Using all the features, two traditional classification models neglect network information resides in contact tracing data and have lower AUC scores (Logistic Regression has mean AUC 0.73 with CI [0.69, 0.77] and Random Forest achieves mean AUC 0.82 with CI [0.78, 0.86]) than GCN. Instead, the GCN model utilizing the network structure has the best performance (AUC: 0.84; CI: 0.80 ~ 0.88). The performance improvement of GCN after adding ‘Feature Set 2’ to ‘Feature Set 1’ is negligible. This suggests that GCN effectively utilized the network structure information contained in ‘Feature Set 1’, thus the additional information from ‘Feature Set 2’ is not significant. GCN inherently incorporates network structure information, while LR and RF do not directly utilize this information unless it is explicitly included in the feature sets 2 and 3 (e.g., the number of cases among neighbours is similar to node degree). Details of model parameters and training details of the three models are presented in the Supplementary Materials. For each contact in the contact tracing network, GCN can leverage the feature information of all neighbours, including those connected by an edge and those connected through two edges, by utilizing two graph convolution layers. This capability allows GCN to use not only direct neighbours’ information but also information from neighbours of neighbours. Although Feature Set 2 and Feature Set 3 provide summarized neighbourhood information – such as the number of cases among neighbours, the maximum age of cases, the most severe level of cases, the number of contacts due to living together, and the number of contacts due to dining together – GCN can directly incorporate this information through its graph convolution mechanism. Consequently, the performance improvement of GCN is the best compared to other models, even when all feature sets are utilized.

Table 1 shows the results for different models under different input features. GCN could utilize the network structure information, which is helpful in the community transmission scenario, and the performance is the most robust across different feature sets as input.

**Figure 6.**
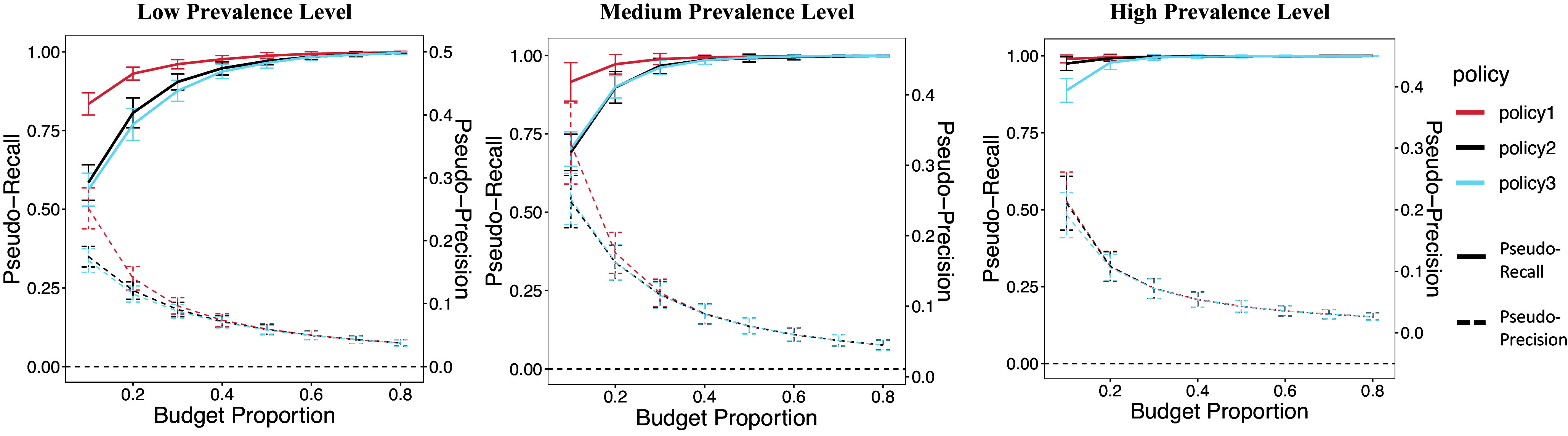
This shows the performance of three policies in Pseudo-Recall and Pseudo-Precision under different prevalence levels.

We then use the risk scores retrieved by the GCN model to select the contacts. We further evaluate the precision and recall of different screening policies based on personalized risk scores under different scenarios of budget proportions and prevalence levels. The budget proportion is defined as the ratio of the number of traced contacts who were tested to the total number of traced contacts. PseudoPrecision shows how reliable a positive result of the screening policy is, while PseudoRecall indicates how effectively the testing policy identifies true positives. We find out that the larger the budget proportion is, the higher the Pseudo-Recall and the lower the Pseudo-Precision is. As shown in Figure 6, different testing policies have relatively similar performance for contacts in a high-prevalence level district (the infected proportion is from 0.75 to 1.0). As for the medium-prevalence level (the infected proportion is from 0.25 to 0.75) and the low-prevalence level district (the infected proportion is from 0.00 to 0.25), the testing policy of selecting contacts with the highest personalized risk scores (Policy 1) is best in terms of Pseudo-Precision and Pseudo-Recall.

## Validation of the current model

In real applications, we obtain the data sequentially. Thus, we split the contact nodes into training, validation, and testing according to the contacts’ quarantine date. Contacts quarantined between 2020-01-02 and 2020-02-04 are used for training; contacts quarantined between 2020-02-05 and 2020-02-10 are used for validation, and contacts quarantined between 2020-02-10 and 2020-07-29 are used for testing. We use the stepwise method [21] to select a combination of features that has the best performance for each model. Then, we train each model by those selected features on the data in the early stage, validate the model in the mid stage, and test the model in the late stage. In Table 2, we list the performance of the three methods. We can see that the GCN still performs the best in validation. More results about the validation of our personalized risk scores are given in the Supplementary Materials.

**Table 2.** The mean AUC scores using the different prediction models

	GCN	LR	RF
AUC-ROC	0.88(0.86 ~ 0.90)	0.77(0.74 ~ 0.80)	0.83(0.80 ~ 0.86)

## Discussion

Based on the network structure, we originally analyse the contact tracing network data by a deep learning method, GCN, to predict the personalized risk scores. The GCN model assumes that two contacts who have had contact before could have similar testing results [17]. In addition, the attribute of each contact in the network also provides some information for predicting the testing results. For example, if we only predict the results using network structure and demographic information, the prediction results are still good. This coincides with prior knowledge: age is highly correlated with testing results [22]. Our model does not give a hard classification. Instead, each person has a personalized risk score of being positive. Based on the risk scores, the government could consider adopting local contact tracing policies to save resources and suppress the spread of pandemic effectively [6]. Thus, we propose several adaptations of policies based on the personalized risk scores. The first two policies are based on risk scores only. The third policy borrows information on the local prevalence level. We apply these policies to screening positive cases, like testing selected contacts, tracing their contacts, or even isolating them. We further evaluate the policies regarding the cost (Pseudo-Precision) and the gains (Pseudo-Recall). The higher the Pseudo-Recall is, the fewer positive cases escape from the screening. The higher the Pseudo-Precision is, the fewer resources are wasted in screening.

To fully consider all the scenarios the governments might have, we observe how our policies perform under varying prevalence levels in the district. Under the three prevalence levels, the first policy outperforms the other two policies in terms of both Pseudo-Recall and Pseudo-Precision. The second policy samples the points based on risk scores, and some more-risky samples might be left out, and some less-risky ones might be included. The third policy estimates the prevalence level locally, which is retrieved from the proportion of cases in the component and is biased. In addition, the estimated prevalence level is no better than the personalized risk score we predicted in distinguishing the two kinds of testing results. Thus, directly selecting the highest risky persons by screening policies is the best solution in terms of both resources and efficiency. However, the difference between the three policies is shrinking as the prevalence increases.

Our study was conducted under conditions where comprehensive contact tracing was still feasible. However, we acknowledge that during periods of medium-to-high prevalence, healthcare systems often face significant resource constraints that can lead to both reduced testing coverage and delayed testing results. This could impact the real-world performance of our risk prediction model in several ways: (1) missing contacts due to incomplete tracing could introduce selection bias, (2) delayed testing results could reduce the model’s utility for timely interventions, and (3) the proportion of undetected cases might increase substantially during high-prevalence periods. To address these limitations, we recommend future research to (1) evaluate model performance under various levels of contact tracing coverage using simulation studies, (2) assess the impact of reporting delays on prediction accuracy, and (3) develop methods to adjust for missing data in high-prevalence scenarios. While these limitations affect the generalizability of our findings to high-prevalence periods, the risk prediction framework we developed remains valuable for early outbreak phases and for regions maintaining robust surveillance systems.

For validation, as the field study of population-based contact tracing is time-consuming and resource-consuming, we could hardly get external datasets to evaluate our policies. Thus, we divide the original dataset sequentially and use the data in the early stage to train, in the middle stage to validate, and in the late stage to test. As a result, GCN still outperforms other methods in the validation stage.

However, our research still has some limitations. One potential limitation of our contact tracing data is the use of symptom onset date metric to classify cases and contacts [23]. The available classification of cases and contacts may not always align with the ground truth due to the possibility of an asymptomatic stage following infection. In addition, some risk score prediction methods use the external dataset to validate [24]. Our model used internal validation instead. Currently, our algorithm is based on contact tracing data collected within only six months. Thus, in the future, our method could be validated on a broader dataset, both in time and in location. Furthermore, if we regard the data as streaming, we could develop a dynamic model framework to update the model based on incoming data and make predictions under the framework of Graph Attention Networks [25]. In addition, our model is a deep learning model, and we could not explain each feature’s contribution as statistics-based models. In the future, we could use some explainable deep learning methods to improve the prediction results and interpret each predictor. In this way, our method could be more transparent and explainable.

Our findings demonstrate that contact tracing data can significantly enhance our understanding of personalized risk prediction. While widespread COVID-19 contact tracing has largely been discontinued, the analytical framework we developed has broad applications for future outbreak response. Specifically, our risk scoring model achieved 84% accuracy in identifying high-risk contacts, suggesting that targeted contact tracing – even with limited resources – can effectively guide public health interventions. For emerging respiratory infectious diseases, we recommend a hybrid approach: combining focused contact tracing in high-risk settings (such as healthcare facilities and congregate living spaces) with complementary data sources like anonymized mobility patterns and social network analyses. This adaptive strategy would enable public health agencies to maximize surveillance impact while operating within resource constraints. Furthermore, our machine learning methodology for risk prediction and screening can be readily adapted to incorporate new data streams and respiratory infectious disease, providing a flexible framework for future outbreak investigations.

Our study period spans from January 2020 to July 2020, during which rapid antigen tests (RATs) were not yet widely available. RATs began to be integrated into public health measures around mid-2020 as a convenient and rapid diagnostic tool, particularly for early screening of asymptomatic cases or during mass testing initiatives. After our study period, RATs became widely available and provided an alternative for screening close contacts. There was a notable change in social distancing policies in April 2020 due to a decline in the number of COVID-19 cases. The government reopened many public venues. Notably, the ratio of untested contacts dropped significantly – from 38% before April 1 to 4% after – likely reflecting reduced tracing during lockdown when fewer positive cases led to fewer contacts being traced. Despite this significant policy change, contact tracing patterns remained largely unchanged, as detailed in the “More Results” section of the Supplementary Materials. In our data pre-processing, we removed several records where the contacts’ age was erroneously recorded above 169. Apart from these few age-related errors, there was no missing data on contact time and type, demographics, neighbourhood information, SARS-CoV-2 test results, or symptom severity levels. We acknowledge that this level of data completeness may not be representative of all settings and datasets, and we encourage researchers working with different data sources to consider the potential effects of missing information in their analyses.

## Supporting information

10.1017/S0950268825100319.sm001 Wu et al. supplementary material Wu et al. supplementary material

## Data Availability

Code for data investigations and simulations is publicly available on https://github.com/SavannahWu99/Personalized-Risk-Score-Prediction.

## Supplementary material

The supplementary material for this article can be found at http://doi.org/10.1017/S0950268825100319.
