# Personalized risk score prediction and testing policy adaptations of a COVID-19 population-based contact tracing network – CORRIGENDUM

**DOI:** 10.1017/S0950268826101976

**Published:** 2026-09-14

**Authors:** Shushan Wu, Yan Feng, Huimin Cheng, Hui Huang, Yang Li, Feng Ling, Ping Ma, Wenxuan Zhong, Ye Shen

When this article was published in Epidemiology & Infection it included some funding information in error. This has since been corrected.

The authors apologise for this error.
