# Supplementary material for: Personalized risk score prediction and testing policy adaptations of a COVID-19 population-based contact tracing network
Source: Epidemiol Infect. 2025 Jul 24;153:e90. doi: 10.1017/S0950268825100319 (PMC12394023; doi:10.1017/S0950268825100319)
Supplement: Wu et al. supplementary material [file S0950268825100319sup001.zip › Appendix_Covid.docx]

# Appendix

## Overview of the Dataset

The dataset a population-based cohort of 827 index patients infected with SARS-CoV-2 and their 14814 close contacts under contact tracing surveillance program from Jan 2020 to July 2020 from a province in eastern China.

The field workers documents information of each contact event. For each contact event, the field workers document the demographics of the two subjects who have contacted, respectively, age and gender, the PCR testing results, the severity level of the tested positive subjects, and information about means of contact. In the contact tracing program, one subject could appear in different contact events, which means that the subject has contacted several subjects during the period.

#### Definition of Severity Level of COVID-19

The field study defines three severity levels of COVID-19, mild, moderate, and severe, at the time of diagnosis. The mild level should have very mild clinical symptoms with no abnormality in radiology of the lungs. The moderate level should have some clinical symptoms, such as fever, coughing, and other respiratory symptoms, and radiology scan shows pneumonia. The severity level definition is different for adults and children.

#### Contact tracing protocol

Contacts were defined as individuals who had direct or indirect interactions with confirmed COVID-19 cases. The interactions could include living together, studying together, working together or other types occurring in enclosed areas, as well as contact involving healthcare providers such as doctors and nurses. Health professions from the local Centers for Disease Control and Prevention (CDC) identified and traced contacts using various methods. Epidemiological investigations were conducted to gather detailed information about the contact events, as well as basic demographic information for both the confirmed cases and their contacts. We categorized the contact events into eight distinct categories, which are defined as follows:

1) Conversion: Individuals having any conversation with a diagnosed and confirmed Covid-19 patients

2) Dine Together: Individuals who dined with the Covid-19 patient (either outdoors or indoors)

3) Enclosed Space Without Direct Contact: Individuals who stayed with the Covid-19 patient in an enclosed area (e.g., classroom, office, elevator, etc.) without conversation or direct contact

4) Healthcare Setting: Healthcare professions who provided healthcare services to a Covid-19 patient

5) Live Together: Individuals who lived with the Covid-19 confirmed patient as a family member, relative, or roommate, etc.

6) Multiple: Individuals who contacted the Covid-19 patient in more than one way, such as living together, eating together, and shared transportation.

7) Shared Transportation: Individuals who commuted with Covid-19 patients, including in trains, cars, airplanes, ships, etc.

8) Others: Other scenarios including but not limited to living in a community or food delivery, etc.

Close contacts were quarantined for at least 14 days, either centrally or at home if resources were limited. Quarantine continued even with a negative COVID-19 test. Health professionals monitored symptoms daily, and RT-PCR tests were administered if respiratory symptoms arose or if a physician suspected infection. If a contact tested positive, contact tracing was initiated for their contacts. Those who remained symptom-free or tested negative were released after 14 days.

#### List of Variables Used

**Demographics characteristics:** The table documents the characteristics of two demographic features from different age and gender groups, of the of seed cases (index cases and additional cases), and close contacts that are excluded the seed cases.

| Characteristics | Group | Index Cases (827) | Close Contacts (14814) |
| --- | --- | --- | --- |
| Age | <20 | 25 | 1782 |
|  | 20-29 | 59 | 1831 |
|  | 30-39 | 146 | 2722 |
|  | 40-49 | 175 | 2618 |
|  | 50-59 | 194 | 2489 |
|  | 60-69 | 97 | 1361 |
|  | $\geq$70 | 52 | 947 |
| Gender | Female | 427 | 6936 |
|  | Male | 400 | 7878 |
| PCR Testing Results | Positive | 827 | 275 |
|  | Negative | 0 | 8593 |
|  | Not tested | 0 | 5946 |

*Table A.1: The summary statistics of the demographic characteristics of groups, index cases, and their close contacts.*

**Neighbors’ information:** for each node in the contact tracing network, we get the statistics of their neighborhood information. The neighborhood of each node in the contact tracing network is defined as nodes that are connected to the node by an edge, which means, the subjects that have directly contacted the person during the period. The number of cases among the neighbors of every person in the contact tracing program is defined as the total number of tested positive subjects among all the contacts. The maximum age of cases among neighbors is defined as the maximum age of all the neighbors of the subject. As for most severe level of cases among neighbors, we first convert the severity level into a ordinal variable, negative subjects’ severity levels are 0, positive subjects have severity levels 1, 2, 3, corresponds to mild, moderate, and severe conditions, respectively. Among the neighbors of a subject in the contact tracing program, the most severe level of the cases among neighbors would be 0 if the neighbors are all PCR tested negative.

| Characteristics | Statistics | Index Cases (827) | Close Contacts  (14814) |
| --- | --- | --- | --- |
| The number of cases among neighbors | Mean | 1.00 | 1.03 |
|  | Max | 1.00 | 4.00 |
|  | Min | 1.00 | 1.00 |
| The maximum age of cases among neighbors | Mean | 63.98 | 45.87 |
|  | Max | 106.00 | 96.00 |
|  | Min | 1.00 | 1.00 |
| The most severe level of cases among neighbors | Mean | 2.33 | 2.28 |
|  | Max | 3.00 | 1.00 |
|  | Min | 1.00 | 3.00 |

*Table A.2: The detailed statistics of the neighbors’ information features*

**Contact type information:** For each contact event, the field workers also document the means of contact, for each subject, we summarize the contact information of the neighbors and make it the feature of the subject, which can indicate the activity level of the subjects. There are major contact types we used as representative, living together and dining together. The two contact types have longer contact time compared with other contact types, and subjects contacted through such means would be more susceptible to be infected. Thus, we use the number of contacts through living together and number of contacts through dining together as the contact type information feature for each subject in the contact tracing program.

| Characteristics | Statistics | Index Cases (827) | Close Contacts (14814) |
| --- | --- | --- | --- |
| The number of contacts through living together | Mean | 2.59 | 0.14 |
|  | Max | 16.00 | 4.00 |
|  | Min | 0.00 | 0.00 |
| The number of contacts through dining together | Mean | 3.07 | 0.17 |
|  | Max | 288.00 | 4.00 |
|  | Min | 0.00 | 0.00 |

*Table A.3: The statistics of the features about contact type information.*

#### Network data

Contact tracing program inherently consists of a network, where each node represents a subject in the program, and the edge connecting two nodes represent two subjects have contacted during the period. Each node in the network can have a feature vector of the demographics, neighbors’ information and contact type information of neighbors. In addition to that, the PCR testing results is the label for each node, positive or negative. The contact tracing network is started from seed nodes, which are tested positive at the beginning.

The subjects can be grouped into several connected components in the contact tracing network, where the subjects in one connected component does not have any direct or indirect contact with another connected component. This phenomenon is reasonable since the subjects in the contact tracing program could have different living circles and a group of subjects could never find a connection to the subjects in another group. To predict the personalized risk scores of each subject, we assume the subjects in one connected component cannot inform the testing results of the other connected component.

## Algorithm

#### Generation of Simulated Data

The contact tracing data can be built into a network with 653 connected components. For each connected components, we can calculate the prevalence level, the ratio of total number of positive subjects in the component to the total number of subjects in the components. We category the prevalence into three groups:

- Low prevalence level: 0~0.25
- Medium prevalence level: 0.25~0.5
- High prevalence level: 0.5~1

Among 653 connected components, there are 320 with low prevalence level, 137 with medium prevalence level, and 69 with high prevalence level. The connected components with medium and high prevalence levels are scarce, we resample the components to make the dataset evaluated more balanced. To emulate the contact tracing data, we use the idea of bootstrap to resample the connected components of the contact tracing network and increase the proportion of connected components with specific prevalence levels. For each prevalence level, the resampling proportion is:

- Simulated network with low prevalence level: resample the 550 connected components with low prevalence levels, 20 with medium prevalence levels, and 20 with high prevalence levels.
- Simulated network with medium prevalence level: resample the 20 connected components with low prevalence levels, 800 with medium prevalence levels, and 50 with high prevalence levels.
- Simulated network with high prevalence level: resample the 10 connected components with low prevalence levels, 40 with medium prevalence levels, and 500 with high prevalence levels.

#### Policy Information

Given the predictions of personalized risk score, we can design three policies for screening out contacts to take PCR tests.

- The first policy is to screen out contacts with the highest risk scores given budget proportion;
- The second policy would use personalized risk scores to generate probabilities to sample close contacts, and the sampling probability of the contact$i$ is:

$$P_{i}=\frac{Z_{i}}{\max_{i} \left( Z_{i} \right)-\min_{i} (Z_{i})};$$

- The third policy considers the local area's prevalence level, generating probability combining the prevalence level of components, $R_{i}$and risk scores $Z_{i}$, and the sampling probability of the contact$i$ is:

$$\tilde{P}_{i}=\frac{R_{i}Z_{i}}{\max_{i} \left( {R_{i}Z}_{i} \right)-\min_{i} (R_{i}Z_{i})}$$

where $R_{i}$ is the prevalence level of the component where $i$-th contact resides.

## More Results

Figure 7 shows t-test results of two risky factors, the left one is local prevalence level. The right one is personalized risk score. We could see that the risk score’s difference between two groups (positive & negative) is more significant than local prevalence level’s difference.

*Figure 7: This shows t-test results of two risky factors, the left one is local prevalence level. The right one is personalized risk score. We could see that the risk score’s difference between two groups (positive & negative) is more significant than local prevalence levels.*
